# Immunosuppression-induced clonal T-cell lymphoproliferative disease causing severe diarrhoea mimicking coeliac disease following renal transplantation: a case report

**DOI:** 10.1186/s12882-020-01884-9

**Published:** 2020-06-10

**Authors:** Emily K. Glover, R. Alexander Speight, Despina Televantou, Stephanie Needham, Neil S. Sheerin

**Affiliations:** 1 Translational and Clinical Research Institute, Newcastle University, Newcastle upon Tyne, NE2 4HH UK; 2 Renal Services, Newcastle upon Tyne Hospitals NHS Foundation Trust, Newcastle upon Tyne, NE7 7DN UK; 3 Department of Gastroenterology, Newcastle upon Tyne Hospitals NHS Foundation Trust, Newcastle upon Tyne, NE1 4LP UK; 4 Department of Pathology, Newcastle upon Tyne Hospitals NHS Foundation Trust, Newcastle upon Tyne, NE1 4LP UK

**Keywords:** Transplant, Lymphoproliferation, Lymphoma, Diarrhoea, PTLD

## Abstract

**Background:**

Post-transplant lymphoproliferative disease is a recognized complication following solid organ transplantation. This is usually a B cell disease and frequently associated with Epstein Barr virus infection, although T cell PTLD can occur. T cell PTLD is usually a monomorphic, lymphomatous disease associated with an adverse prognosis.

**Case report:**

We report a 52 year old male pre-emptive renal transplant recipient who developed severe diarrhea with weight loss following intensification of his immunosuppression due to antibody mediated rejection 3 years after transplantation. Duodenal biopsy demonstrated monoclonal CD8+ T cell duodenitis leading to increased intraepithlieal lymphocytes and sub-total villous atrophy mimicking coeliac disease. Coeliac disease was excluded by negative anti-tissue transglutaminase antibody, HLA-DQ2 and HLA-DQ8 testing. There was no evidence of lymphoma either on biopsy or CT enterography and no FDG avid disease on PET. Symptoms did not improve with reduction of immunosuppression, but resolved fully on complete withdrawal of treatment. The transplant failed and he was established on dialysis. The diagnosis was early PTLD.

**Conclusions:**

Oesophagogastroduodenoscopy with small bowel biopsies is a useful investigation for determining the cause of diarrhoea in renal transplant patients when more common causes have been excluded. This is the first report that we are aware of clonal T cell PTLD mimicking coeliac disease which only resolved after complete withdrawal of immunosuppression. As treatments for lymphoma are aggressive they are only initiated in the malignant phase and management of early stage PTLD is to minimise risk of progression by reducing immunosuppression. Any plans to retransplant will have to take into consideration the possibility that PTLD will recur.

## Background

The term post-transplant lymphoproliferative disease (PTLD) was first introduced in 1984 [1] and describes a range of pathologies occurring as a consequence of immunosuppression from reactive lymphoid hyperplasia to lymphoma. The incidence of PTLD after kidney transplantation is ~ 3% [2] and is increased by more intensive immunosuppression. PTLD can be either monomorphic or polymorphic and B cell PTLD is frequently associated with Epstein Barr virus (EBV) infection or reactivation with cells showing a distinct pattern of EBV antigen expression. T cell PTLD is less common and accounts for 4% of cases in the early post-transplant period and 15% of cases of late PTLD (> 2 years since transplant) [3]. T cell PTLD is a heterogenous group of diseases which are only associated with EBV infection in around 30% of cases [4, 5]. These are most commonly monomorphic, although polymorphic disease is described, and associated with a poor prognosis.

In patients with renal allografts there are many potential causes of persistent diarrhoea, including those associated with immunosuppression, such as the side effects of mycophenolic acid and intestinal PTLD, and those that cause diarrhoea in non-immunosuppressed patients. Coeliac disease affects around 1% of adults, can affect transplant recipients and should be included in the list of differential diagnoses. Coeliac disease, due to an immune response to dietary gluten, typically causes gastrointestinal symptoms, but is also associated with a range of non-GI manifestations. Autoantibodies to tissue transglutaminase (anti-TTG) are found in 95% of patients with coeliac disease but duodenal biopsy remains the cornerstone of diagnosis. Typically, this shows evidence of a lymphocytic infiltrate, crypt hyperplasia, inflammation of the lamina propria and villous atrophy [6]. Treatment is with a gluten free diet, although rarely refractory disease is recognised.

We report a patient who developed severe diarrhoea with a T cell rich lymphocytic duodenal infiltrate, mimicking coeliac disease, but due to monoclonal T cell PTLD. The disease resolved on withdrawal of immunosuppression.

## Case presentation

A 52 year old man received a pre-emptive renal transplant from his wife for the treatment of end stage renal disease due to autosomal dominant polycystic kidney disease (HLA mismatch 1–1-1, CMV mismatch D+/R-, EBV IgG not detected). He received alemtuzumab at induction (30 mg subcutanously on day 0 and day 1) followed by maintenance immunosuppression with tacrolimus, mycophenylate mofetil (MMF) and prednisolone. As he was enrolled in a clinical trial, the use of alemtuzumab for induction was determined by the arm he was randomised to. He continued on tacrolimus before being randomised at 6 months to switch to sirolimus as part of the clinical trial [7]. A year later he had to leave the study as he was undergoing incisional hernia repair so returned to tacrolimus-based immunosuppression.

Three years after transplantation renal function started to decline. Chronic antibody mediated rejection (CAMR) was found on transplant renal biopsy and new donor specific antibodies against HLA class II were detected. Tacrolimus and MMF doses were increased and he restarted on 5 mg prednisolone. The daily total tacrolimus dose was increased from 3 mg to 5 mg with levels rising from 5.4 μg/L to 7.0 μg/L and MMF daily dose was doubled from 1000 mg to 2000 mg. A month after this change he presented to primary care having passed frank blood in his stool and with a one week history of abdominal pain and faecal urgency. In response to these symptoms MMF was switched to mycophenolic acid, but the diarrhoea continued and he was admitted on two occasions due to worsening renal function.

Five months after biopsy confirmed CAMR, rituximab was started at a dose of 200 mg with a planned frequency of every 6–8 months and mycophenolic acid was withdrawn. Although rectal bleeding resolved the diarrhoea continued and he experienced 4 kg of weight loss over 2 months with an overall weight loss of around 14 kg over a year.

Investigations for the cause of his diarrhoea included CMV and EBV PCR and stool culture which were all negative at initial presentation. Faecal elastase was also normal and gastric parietal cell and enterocyte antibody testing was negative. Flexisigmoidoscopy showed non-bleeding haemorrhoids and colonoscopy was normal. Radionucleotide SeHCAT bile study to assess for bile acid malabsorption was also normal. Imaging studies including PET, CT thorax-abdomen-pelvis, CT enterography and MRI small bowel showed no evidence of malignancy or other bowel pathology.

EBV DNA was not detectable in serum by PCR at the onset of diarrhoea and EBV IgG remained negative. However, 6 weeks after the onset of diarrhoea EBV DNA was detected at titres of 1612 IU/ml and then fluctuated between not detected and 6770 IU/ml for the duration of his diarrhoea. EBV DNA was detected at low levels (< 1000 IU/ml) when tacrolimus dose was further reduced (levels in the region of 3.8 μg/L). Given symptomatic improvement EBV DNA PCR was not repeated on complete withdrawal of immunosuppression.

**Fig. 1:**
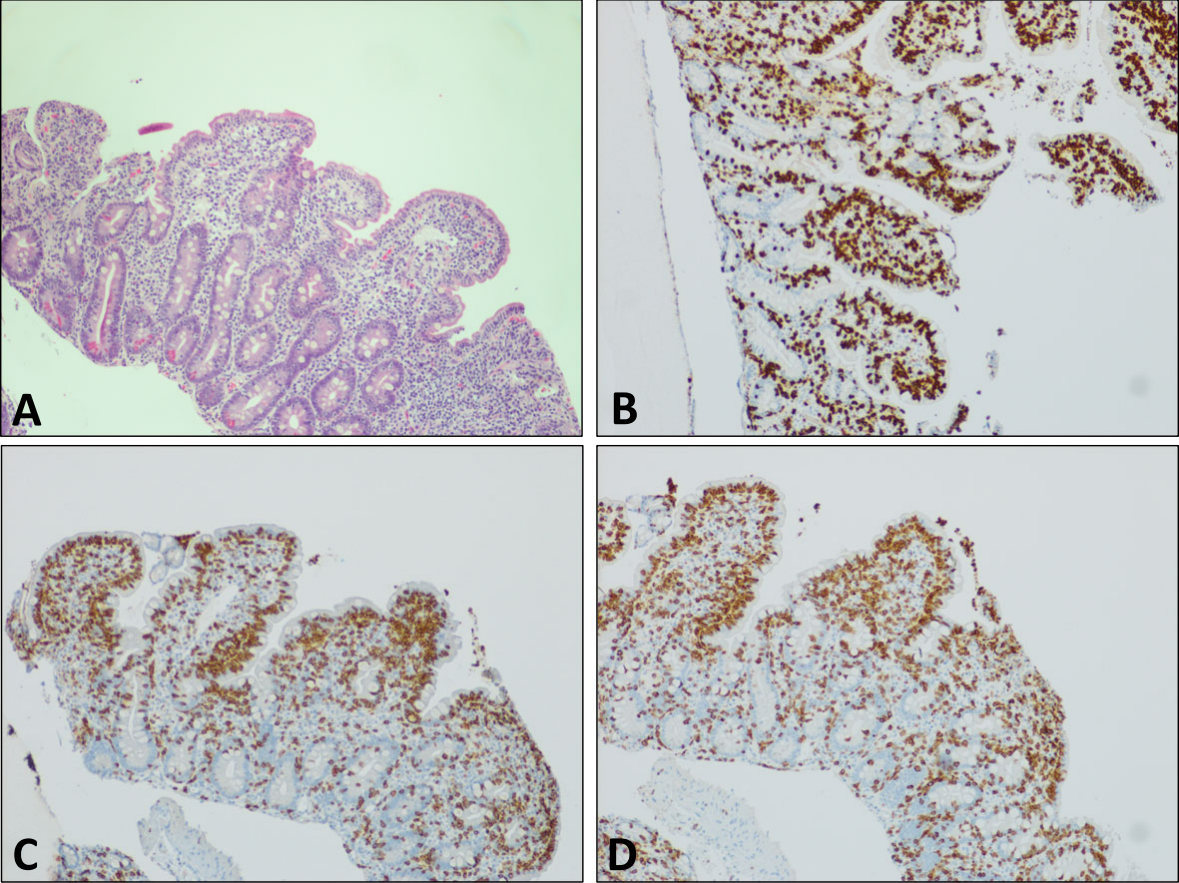
Duodenal mucosa showing subtotal villous atrophy, crypt hyperplasia and increased intraepithelial lymphocytes on (**a**) H&E staining. Immunohistochemical staining (shown in brown) of duodenal mucosa for (**b**) TCR, (**c**) CD8 and (**d**) CD3. All images × 10 magnification

Creon was started under gastroenterology advice and loperamide was prescribed for symptomatic relief. Oesophagogastroduodenoscopy (OGD) was performed to investigate loss of appetite and further weight loss. Biopsies from the duodenum identified moderate lymphocytic duodenitis with mucosal damage as evidenced by shortening villi, in keeping with coeliac disease *(*Fig. 1*)*. However, diarrhoea was unresponsive to a gluten free diet and he was negative for antibodies to tissue transglutaminase and HLA DQ2 and DQ8.

Repeat OGD after 6 months of gluten free diet identified a clonal T-cell population. The intra-epithelial T-cells had normal immunohistochemistry, in that they expressed the antigens CD3 and CD8, but reproducible clonal T-cell receptor (TCR) beta and TCR gamma rearrangements were detected using a multiplex PCR assay [8]. Immunoglobulin heavy chain gene rearrangements were polyclonal, excluding clonal B cell proliferation. There was no evidence of lymphoma either on biopsy or CT enterography and no FDG avid disease on PET.

After further reductions in tacrolimus repeat duodenal biopsies found a persistence of a clonal T cell population and mucosal damage with subtotal villous atrophy and a suggestion of crypt hyperplasia. Almost 3 years since the episode of CAMR and onset of diarrhoea all immunosuppression was withdrawn and peritoneal dialysis was commenced. The diarrhoea improved within days of stopping immunosuppression and the patient remains well on dialysis with no diarrhoea and he has successfully regained the weight he lost when unwell.

## Discussion and conclusions

We report duodenitis with seronegative villous atrophy and non-malignant clonal T-cell proliferation secondary to immunosuppression in a renal transplant recipient. The diagnosis of early PTLD is supported by full resolution of symptoms on withdrawal of immunosuppression. As far as we are aware there are no other case reports describing this set of findings.

Lymphoma, including PTLD, is a recognised complication following solid organ transplantation. In this case, the clonal T cell proliferation was pre-malignant with a high risk of progressing to enteropathy associated T cell lymphoma (EATL). Our patient had a high immunosuppressive burden at the time of presentation as his therapy had been increased to manage CAMR and he had received alemtuzumab at induction. As a lymphocyte-depleting agent, alemtuzumab causes prolonged immunosuppression with full B cell recovery taking 12 months and T cells reaching 50% of normal levels at 36 months [9]. Treatments for lymphoma are aggressive so they are only initiated in the malignant phase and management of early stage PTLD is to minimise risk of progression by reducing immunosuppression. Histological findings in lymphoma are variable but can include morphological evidence of mucosal damage by an aberrant lymphocytic population with an abnormal lymphocytic phenotype and loss of at least one of the T-cell antigens. On imaging the presence of FDG avid lesions on PET is characteristic [10]. Neither of these features occurred in our case.

Other differentials for seronegative villous atrophy include coeliac disease and autoimmune enteropathy. Autoimmune enteropathy is a rare disease, more common in children, and the diagnosis was not supported here as all antibody testing was negative and the condition is treated with immunosuppression [11].

Coeliac disease that is non-responsive to a gluten free diet and has clonal T-cells on biopsy is termed refractory coeliac disease type II (RCD II). In this condition intraepithelial lymphocytes are CD3+ and CD8- so are phenotypically abnormal and the associated increased risk of transformation to EATL would encourage a reduction in immunosuppression [12]. Anti-TTG testing is important for making the diagnosis of coeliac disease with sensitivity of > 90% and specificity of > 95%. TTG is an enzyme that removes an amide group from gluten peptides to increase their binding to HLA-DQ2 and DQ8 on antigen presenting cells, stimulating an inflammatory response. HLA-DQ2 and DQ8 are strongly associated with coeliac disease as 95% of those with the condition are positive for HLA-DQ2 and the other 5% positive for HLA-DQ8 but 30–40% of healthy individuals will also be positive for one of these alleles. HLA testing is useful to exclude coeliac disease as the negative predictive value approaches 100%. We can be confident that our case was not coeliac disease as he was negative for anti-TTG and HLA-DQ2/DQ8 [13, 14].

Our patient had complete resolution of his symptoms following withdrawal of immunosuppression. Duodenal biopsies have not been repeated, but the assumption is that the duodenitis and villous atrophy will have resolved, mirroring the improvement in symptoms. We do not know whether the T cell clone has been completely destroyed or is now controlled and therefore cannot predict whether the disease will recur if immunosuppression is reintroduced. Patients have been retransplanted after the successful treatment of PTLD [15, 16] but any plans for retransplanting this patient must take into account the possibility that this will recur.

Clonal T cell PTLD is a rare cause of severe diarrhoea in renal transplant recipients. Clinically, it mimics coeliac disease and once more common causes have been excluded, an OGD with small bowel biopsies and immunohistochemistry can be done to look for the clonal T cell population specific for T cell PTLD. As treatment involves withdrawal of immunosuppression there will be implications for the current graft and it may be necessary to trial immunosuppressive drugs for suitable options before considering re-transplantation.

## Data Availability

Data sharing is not applicable to this article as no datasets were generated or analysed during the current study.

## Abbreviations

Anti-TTG: Autoantibodies to tissue transglutaminase
CAMR: Chronic antibody mediated rejection
EATL: Enteropathy associated T cell lymphoma
EBV: Epstein Barr virus
MMF: Mycophenylate mofetil
OGD: Oesophagogastroduodenoscopy
PTLD: Post-transplant lymphoproliferative disease
RCD II: Refractory coeliac disease type II
TCR: T-cell receptor
